# Understanding the Translabyrinthine Approach for Neurosurgery Residents: Operative and Educational Video

**DOI:** 10.1055/a-2780-4133

**Published:** 2026-02-13

**Authors:** Beste Gülsuna, Xiaochun Zhao, Alexander G. Bien, Jeffrey A. Zuccato, Christopher S. Graffeo

**Affiliations:** 1 Department of Neurosurgery, University of Oklahoma College of Medicine, Oklahoma City, Oklahoma, United States; 2 Department of Otolaryngology—Head and Neck Surgery, University of Oklahoma College of Medicine, Oklahoma City, Oklahoma, United States

**Keywords:** vestibular schwannoma, translabyrinthine approach, facial nerve preservation, skull base surgery, intraoperative monitoring

## Abstract

Vestibular schwannomas are benign tumors of the vestibular division of the eighth cranial nerve, with an incidence of 1 to 2 per 100,000 annually. Large tumors (>3 cm) may cause disabling symptoms such as progressive hearing loss, vestibulopathy, or trigeminal nerve dysfunction, often necessitating microsurgical resection. This video demonstrates the translabyrinthine resection of a 3.2-cm left-sided vestibular schwannoma in a 67-year-old woman with worsening sensorineural hearing loss and new-onset lip numbness ( Video 1 ). The procedure was performed at a tertiary center with continuous intraoperative neurophysiological monitoring of cranial nerves V, VI, VII, X, and XI, as well as somatosensory and motor evoked potentials. Near-total resection (∼99%) was achieved, with a small residual adherent to the cisternal segment of the facial nerve near the superior petrosal vein to maximize functional preservation. Postoperatively, the patient experienced transient House–Brackmann (HB) grade 2 facial palsy, which improved within 5 weeks to HB 1, and no nodular enhancement was observed on follow-up magnetic resonance imaging. The translabyrinthine approach provides direct exposure of the internal auditory canal with minimal brainstem retraction, making it particularly advantageous for large tumors in patients with nonserviceable hearing. Beyond its operative illustration, this video emphasizes step-by-step surgical anatomy, technique, and intraoperative decision-making, offering neurosurgery residents an educational framework for understanding indications, technical nuances, and complication avoidance strategies.

**Video 1** Translabyrinthine Approach for Vestibular Schwannoma.

